# Control of the rate of evaporation in protein crystallization by the ‘microbatch under oil’ method

**DOI:** 10.1107/S0021889808024667

**Published:** 2008-08-30

**Authors:** Boris Brumshtein, Harry M. Greenblatt, Anthony H. Futerman, Israel Silman, Joel L. Sussman

**Affiliations:** a Department of Structural Biology, Weizmann Institute of Science, Rehovot 76100, Israel; b Department of Biological Chemistry, Weizmann Institute of Science, Rehovot 76100, Israel; c Department of Neurobiology, Weizmann Institute of Science, Rehovot 76100, Israel; d Israel Structural Proteomics Center, Weizmann Institute of Science, Rehovot 76100, Israel

**Keywords:** microbatch, crystallization under oil, protein crystallography, evaporation

## Abstract

Microbatch crystallization under oil is a powerful procedure for obtaining protein crystals. Using this method, aqueous protein solutions are dispensed under liquid oil, and water evaporates through the layer of oil, with a concomitant increase in the concentrations of both protein and precipitant until the nucleation point is reached. A technique is presented for regulating the rate of water evaporation, which permits fine tuning of the crystallization conditions as well as preventing complete desiccation of the drops in the microbatch crystallization trays.

## Abstract 2

A procedure is presented for controlling the rate of evaporation during ‘microbatch under oil’ protein crystallization.

## 1. Introduction

Microbatch crystallization under oil (Chayen *et al.*, 1990 ▶) is a widely used and robust method for crystallizing proteins. In this method, nanolitre droplets of protein and precipitant are dispensed into the individual wells of a crystallization tray, and are then covered by either liquid paraffin oil or a mixture of paraffin and silicone oils (Chayen, 1997 ▶, 1998 ▶; D’Arcy *et al.*, 2004 ▶, 2003 ▶). Water slowly evaporates from the crystallization drops through the layer of liquid oil, resulting in an increase in the concentrations of both protein and precipitant, which often yields protein crystals.

One of the significant drawbacks of the method is complete desiccation of the aqueous droplet solutions within several weeks after the experiment has been set up. Over-drying often results in the formation of salt deposits, resulting in disintegration of the protein crystals, with a concomitant loss of diffracting power. We describe below a simple procedure that permits control of the rate of evaporation and prevents the drops from drying out.

**Figure 1:**
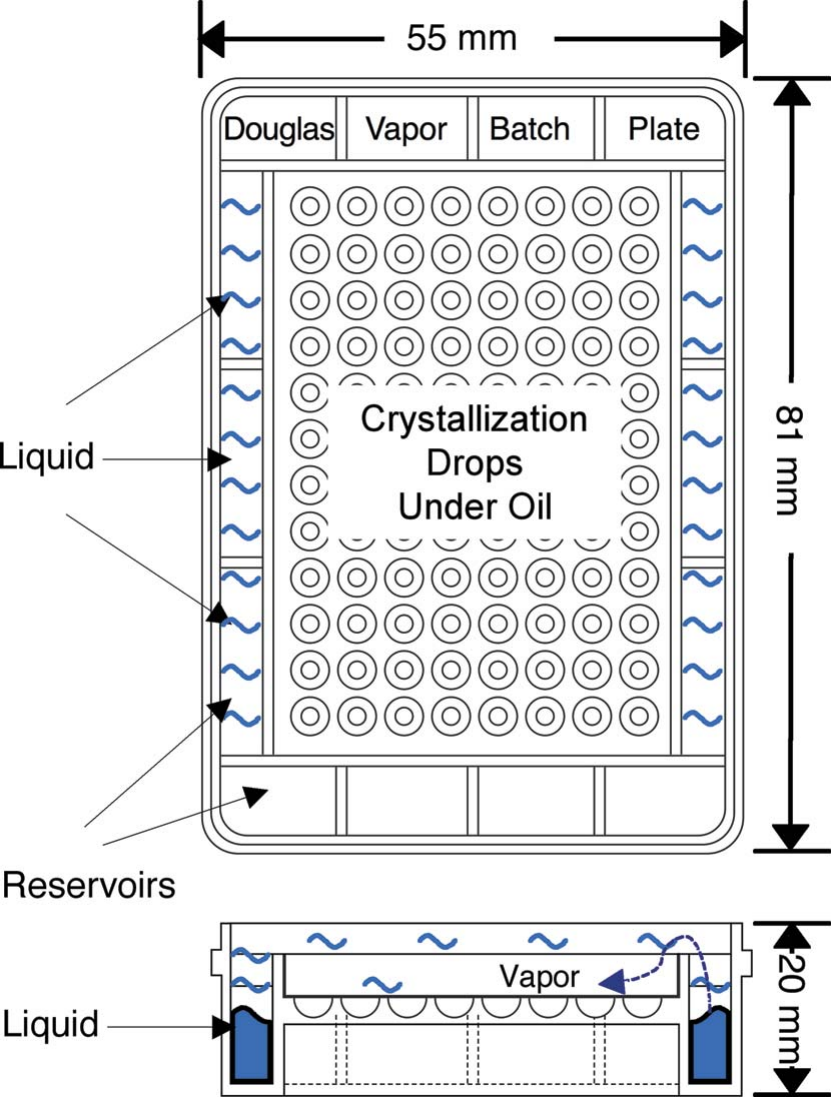
Douglas Vapor Batch hydrophobic crystallization plate. Crystallization droplets are dispensed under oil into the wells in the central part of the plate. The humidity within the tray above the oil-covered droplets is determined by the vapor pressure of the liquid in the reservoirs.

The key innovation is to include an aqueous reservoir within the microbatch crystallization tray (Fig. 1 ▶). By making a suitable choice of the vapor pressure of the solution within this reservoir, it is possible both to control the rate of evaporation from the microbatch droplets and to eliminate the risk of their drying out.

## 2. Methods

### 2.1. Crystallization

Acid-β-glucosidase (Kacher *et al.*, 2008 ▶; Dvir *et al.*, 2003 ▶) was the enzyme chosen for testing the methodology. It is a glycoprotein of molecular weight of ∼60 kDa, with pI = 7.2. The preparation used was a recombinant form of acid-β-glucosidase expressed in plant cells (Shaaltiel *et al.*, 2007 ▶). It was concentrated to 5 mg ml^−1^ in 100 m*M* NaCl/10 m*M* citrate, pH 5.5, containing 7%(*v*/*v*) EtOH. For the crystallization trials, use was made of a 1:1 mixture of the protein solution and of 0.2 *M* Na/KPO_4_/20%(*w*/*v*) PEG 3350, which is one of the components of the Qiagen PACT screen (Qiagen Inc., Valencia, CA, USA). In addition, conditions under which the protein did not tend to crystallize were purposely chosen, so that crystal formation would not impede volume measurements. Crystallization droplets (0.55 µl) were dispensed into Douglas Vapor Batch hydrophobic crystallization plates (http://www.douglas.co.uk) (Fig. 1 ▶) under 4 ml of Al’s oil (1:1 paraffin to silicone oil) (D’Arcy *et al.*, 1996 ▶) making use of an IMPAX 1–5 crystallization robot (http://www.douglas.co.uk).

The plates used contain reservoirs around their perimeters (see Fig. 1 ▶) that could be filled with solutions of a desired composition. The effect of reservoir composition on the rate of concentration of the crystallization droplets was examined using five sets of conditions: (1) An empty reservoir (no liquid at all); (2) 4 ml of distilled water in the reservoir; (3) 4 ml of 0.5 *M* NaCl; (4) 1 *M* NaCl; (5) 2 *M* NaCl.

### 2.2. Measurement of droplet volume

**Figure 2:**
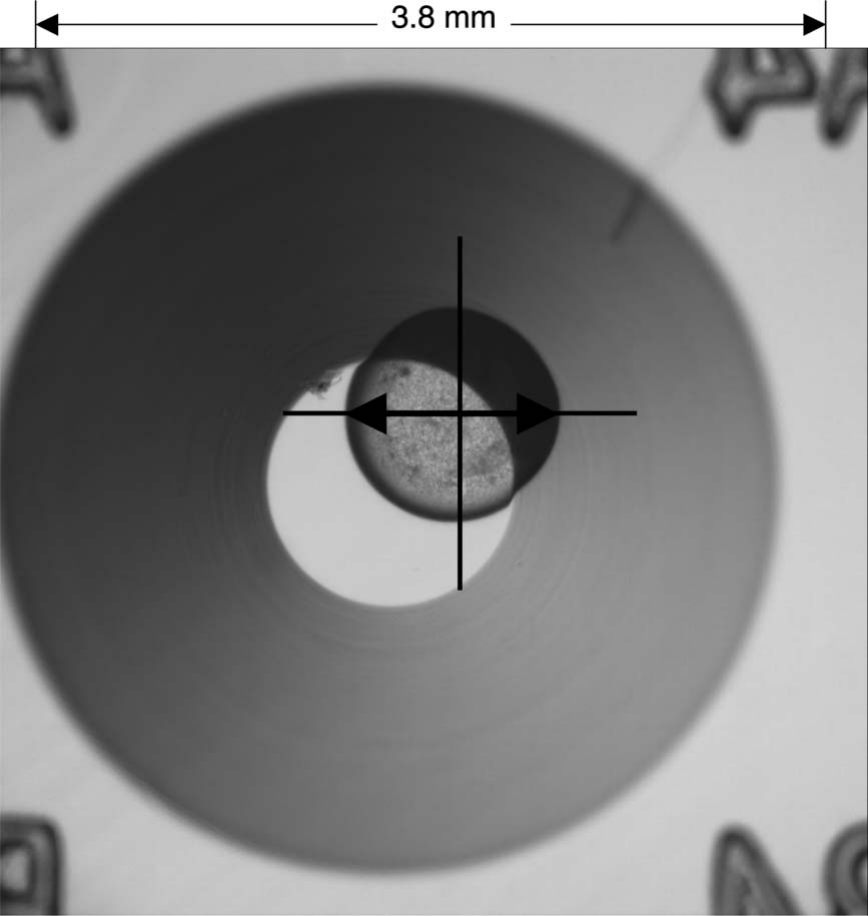
Image of a crystallization droplet under oil in a well in the crystallization plate. The measured diameter of the droplet is indicated by the double-headed arrow.

In each plate, 48 droplets were dispensed. In order to follow evaporation as a function of time, eight droplets at identical locations in all the trays were selected for monitoring. Their diameter was measured using an Olympus microscope equipped with an XR12 lens, which yielded ×90 magnification. The horizontal diameter of the droplet was measured using the microscope scale bar (Fig. 2 ▶), making the assumption that the droplets are spherical. This assumption was shown to be valid by measuring their initial diameters, calculating the volume by use of the formula *V* = (4/3)π*r* ^3^, and comparing this calculated volume with the volume dispensed. Measurements of droplet diameters were made daily for 14 d. The plates were maintained at 295 K and at an external humidity of 65–75%.

## 3. Results

**Figure 3:**
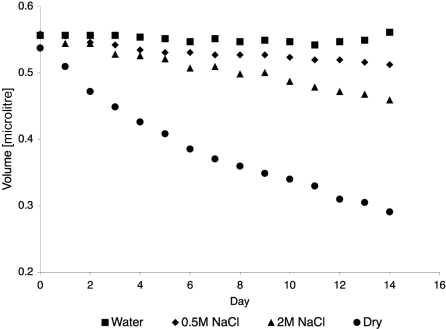
Dehydration of crystallization drops. Volumes of crystallization drops as a function of time. Each point represents the average diameter for eight crystallization droplets. Standard deviations of 1σ are typically below 15% of the calculated volumes (not shown).

The rates of decrease in the calculated volumes of the droplets under the various vapor pressure conditions created by the solutions in the reservoirs are displayed in Fig. 3 ▶. The data clearly show that changing the salt concentration of the aqueous solution in the reservoir, and thereby the vapor pressure, dramatically changes the rate of decrease in the volumes of the droplets. The lower the salt concentration, the higher the vapor pressure, and the lower the rate of decrease in volume. With water in the reservoirs, it was effectively possible to completely prevent concentration of the droplets for periods of as long as two years, under which conditions crystals in the drops maintained their integrity and diffracting power. In some experiments, the trays were wrapped in parafilm, but this had no effect on the rate of evaporation (not shown).

## 4. Discussion

The experimental data presented unequivocally demonstrate that it is possible to control the rate of decrease in volume of the droplets in the microbatch under oil procedure by regulating the vapor pressure within the crystallization tray. This is achieved by adding a salt solution of a desired concentration to the reservoirs around the perimeter of the tray. Although these reservoirs were initially intended to contain an excess of the oil covering the crystallization droplets, they were also shown to be useful for other purposes, such as to serve as reservoirs for 2-propanol in a protocol for crystallizing a retroviral capsid protein domain (Mortuza *et al.*, 2004 ▶).

The technique permits regulation of the rates of crystal formation and growth, since the rate of concentration can be reduced as much as desired by lowering the salt concentration and concomitantly raising the vapor pressure. Another advantage of the technique is the ability to arrest evaporation completely, thus eliminating the risk of desiccation, which can lead to disintegration of the crystals and to consequent loss of diffracting power.

In the experiments in which the reservoirs contained distilled water, condensation of small water droplets (∼0.01–0.05 µl), both within the oil and on the plastic surface, produced an undesirable artefact. It may be due to temperature fluctuations or, more likely, to the high vapor pressure of the distilled water. This artefact can be avoided by the use of a salt concentration as low as 0.5 *M* NaCl.

In summary, the novel methodology presented above provides a way to control the rate of evaporation of solvent from crystallization droplets in the batch under oil procedure, and to avoid drying out of the droplets over prolonged periods.
